# Study on antibiotic susceptibility of *Salmonella* typhimurium L forms to the third and forth generation cephalosporins

**DOI:** 10.1038/s41598-020-59456-8

**Published:** 2020-02-20

**Authors:** Cuiping Yang, Huihui Li, Tao Zhang, Yifan Chu, Junli Zuo, Dengyu Chen

**Affiliations:** 1 Department of Gastroenterology, Ruijin Hospital North, Shanghai Jiaotong University School of Medicine, Shanghai, 201801 P.R. China; 2 Department of Microbiology, Bengbu Medical College, Bengbu, Anhui 233030 P.R. China; 3 Anhui Key Laboratory of Infection and Immunity, Bengbu Medical College, Bengbu, Anhui 233030 P.R. China; 4 Department of Geriatric Medicine, Ruijin Hospital North, Shanghai Jiaotong University School of Medicine, Shanghai, 201801 P.R. China; 5 Laboratory Center for Morphology, Bengbu Medical College, Bengbu, Anhui 233030 P.R. China

**Keywords:** Antibiotics, Bacterial structural biology

## Abstract

*Salmonella* typhimurium is a pathogenic gram-negative bacterium, which is found primarily in the intestinal lumen. It often causes diarrhea in infants and young children and leads to food poisoning. Drug resistance of *Salmonella* typhimurium presented serious complications in clinical patients. In this study, we investigated the antibiotic susceptibility of *Salmonella* typhimurium standard strain L forms to the third and forth generation cephalosporins, in order to control and eliminate *Salmonella* typhimurium L forms in infection treatment. *Salmonella* typhimurium L forms were induced by β-lactam antibiotic cefazolin in the culture medium of bacterial L forms. The antibiotic susceptibility of *Salmonella* typhimurium L forms was analyzed by K-B drug susceptibility testing. The change trend of drug susceptibility and resistance of *Salmonella* typhimurium L forms was obtained in accordance with USA clinical and laboratory standards institute (CLSI) evaluation data and statistical analysis. Drug resistance of *Salmonella* typhimurium L forms showed little increasing trend compared with their parent bacteria. The L form inhibition zone was smaller than in the parent bacteria. However, the drug susceptibility of L forms of *Salmonella* typhimurium to the third and forth generation cephalosporins remained sensitive.The antibiotic susceptibility of *Salmonella* typhimurium L forms to the third and forth generation cephalosporins remains sensitive, and the combined use of multi-antibiotics is a convenient and effective method to reduce *Salmonella* typhimurium L forms occurrence.

## Introduction

Because of the immature immunity of infants and young children, bacterial acquired antibiotic resistance and poor sanitation, *Salmonella* typhimurium (*S*. typhimurium) has become the main pathogen in nosocomial and foodborne infection^1–3^. *Salmonella* typhimurium possesses endotoxin, enterotoxin and extracellular enzymes as well as other pathogenic factors, so that *Salmonella* typhimurium has strong pathogenicity^4,5^. After the use of antibiotics, it was easy to make *Salmonella* typhimurium form into cell wall-defective bacteria named bacterial L forms^6–8^.

BacteriaL L forms still have the ability to cause disease, such as leading to the delay of chronic infection, and decreased susceptibility to antibacterial drugs which act on cell walls^9,10^. In these experiments, we studied the resistance of *Salmonella* typhimurium standard strain L forms to advanced cephalosporins and judged the susceptibility degree of *Salmonella* typhimurium L forms to the third and fourth generation cephalosporins, in order to guide the rational use of clinical antibacterial drugs. The study will provide the basic theory for controlling *Salmonella* typhimurium infection.

## Materials and Methods

### Bacterial strain

*Salmonella* typhimurium standard strain CMCC50115 was purchased from the National Institute for the Control of Pharmaceutical and Biological Products.

### Reagents and instruments

Gram staining solution was purchased from Nanjing Jiancheng Bioengineering Institute. Antibiotic Drug sensitive tablets were purchased from OXOID company, UK, including third-generation cephalosporins, such as cefotaxime (CTX), ceftriaxone (CRO), cefoperazone (CFP), ceftazidime (CAZ), and fourth generation cephalosporin cefepim (FEP). Mueller-Hinton (MH) agar was used for determination of bacterial susceptibility to antibiotics by using agar paper diffusion method. 100 ml MH agar medium was prepared and made with 1.7 g agar, 0.2 g beef extract powder, 0.15 g soluble starch, 1.75 g casein hydrolysate, and afterwards regulating pH 7.4, and autoclaving at 121 °C for 15 min. The MH agar medium was inverted into the plate for antibiotic drug susceptibility testing. Soft agar plates for bacterial L forms culture were prepared by using improved MH agar plates with 1% agar and 2% NaCl. A mounted digital microscope (Nikon, Japan) was used for observing bacterial L forms and taking photos of micrographs.

#### Salmonella typhimurium L form Induction

*Salmonella* typhimurium was induced into stable L-forms by cefazolin drug discs with 30 µg/tablet. Gram stain was used to observe the microscopic morphology of *Salmonella* typhimurium and its L forms.

#### Preparation of Salmonella typhimurium and L form bacterial liquid

The bacteria in the logarithmic growth phase were obtained by conventional bacterial culture. 10^8^ CFU/ml bacterial liquid was prepared with sterile physiological saline to a 0.5 McFarland standard concentration. *Salmonella* typhimurium L forms were subcultured under cefazolin induction, to obtain fresh cultures. 10^8^ CFU/ml L form bacterial liquid was prepared with sterile 3% NaCl hypertonic saline, and counts were measured by McFarland turbidimetry.

### Drug susceptibility test

According to the national clinical test operating protocol for drug susceptibility testing *in vitro*, the first preparation of test broth was made after culturing at 37 °C for 6 hours. The bacterial turbidity degree was regulated to 0.5 McFarland units. The tested bacteria were scribbled and uniform coating on MH agar and agar disc diffusion method was used for drug susceptibility testing. The five kinds of cephalosporin susceptibility paper disc were evenly affixed to the center and surrounding of the plate aseptically. After the conventional bacterial culture, the diameters of the inhibition zones were measured. Referring to the national antibacterial drug susceptibility test criteria, drug susceptibility was determined specifically.

### Statistical analysis

The experiment was repeated three times, and continuous variables are expressed as mean ± standard deviation. Between the two groups the data difference was analyzed by the way of using paired *t* test. A *P* value of < 0.05 was considered statistically significant.

## Results

### Gram staining results

**Figure 1:**
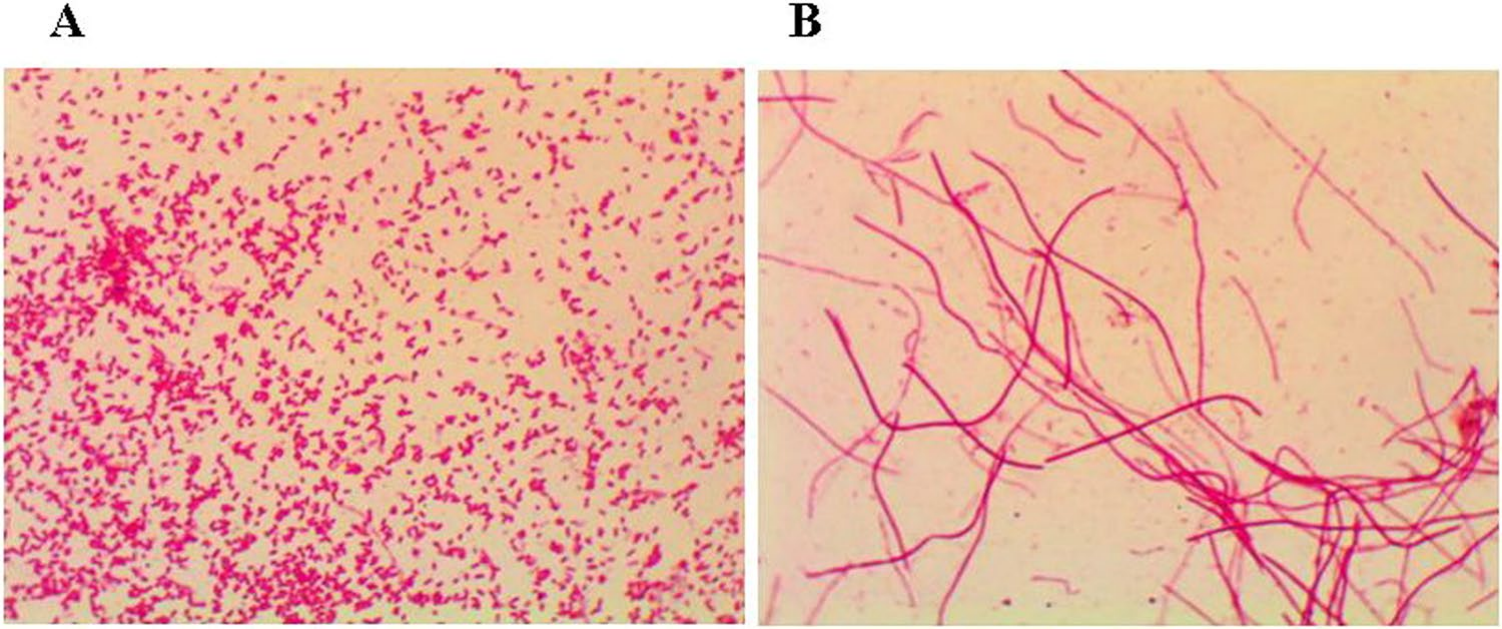
Gram staining morphological observation of *Salmonella* typhimurium and its L forms (Gram stain, magnification: 1000×). (**A**) *Salmonella* typhimurium is negative Gram stain and medium size bacillus. (**B**) *Salmonella* typhimurium L forms are negative Gram stain and filamentous bacillus.

As shown in Fig. 1, the *Salmonella* typhimurium strain was negative Gram stain and medium size bacillus, and *Salmonella* typhimurium L forms were negative Gram stain and filamentous bacillus. *Salmonella* typhimurium L forms were often coiled into groups.

### Susceptibility test results

**Figure 2:**
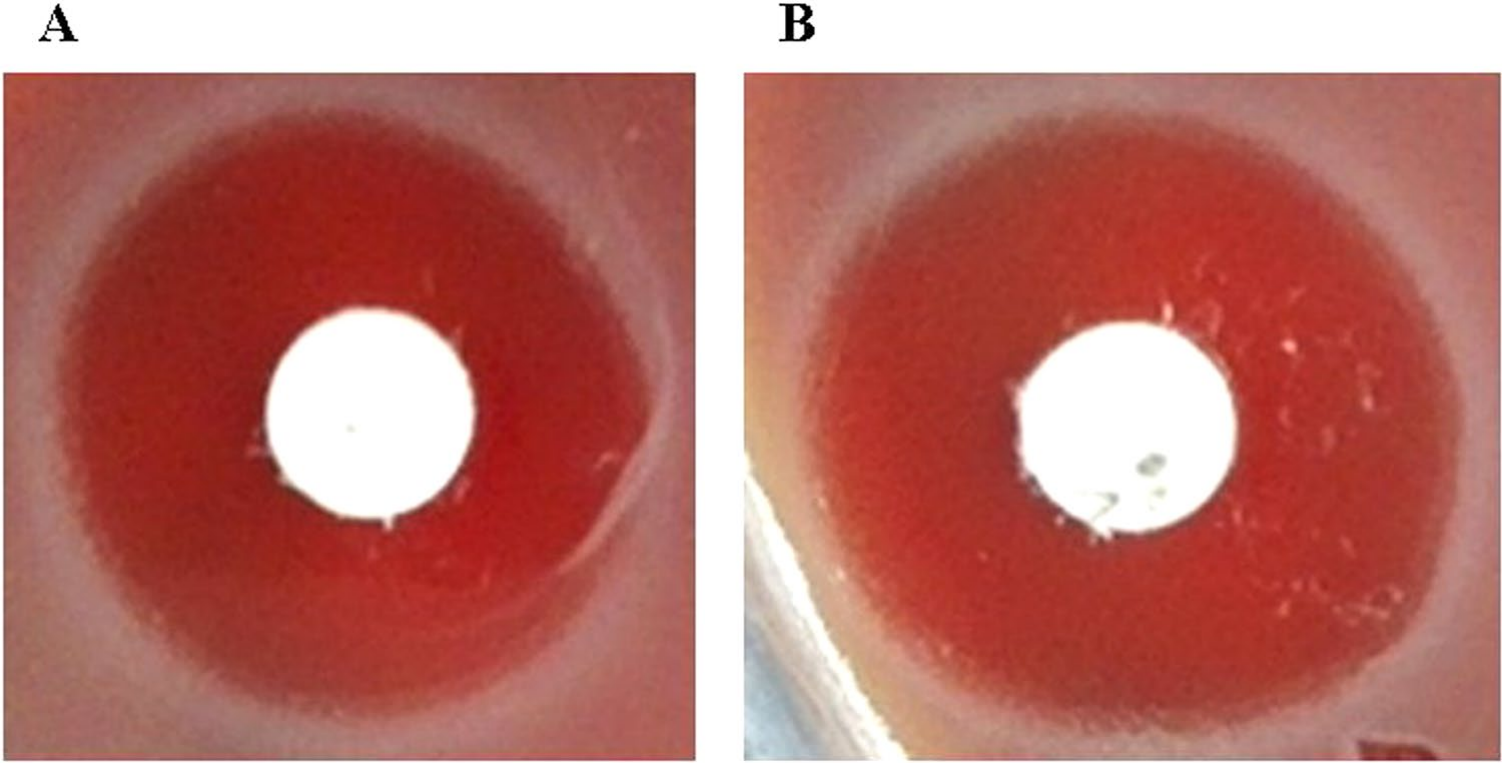
Drug susceptibility test cultures *of Salmonella* typhimurium and its L forms. (**A**) *Salmonella* typhimurium drug susceptibility test culture. (**B**) *Salmonella* typhimurium L forms drug susceptibility test culture.

**Figure 3:**
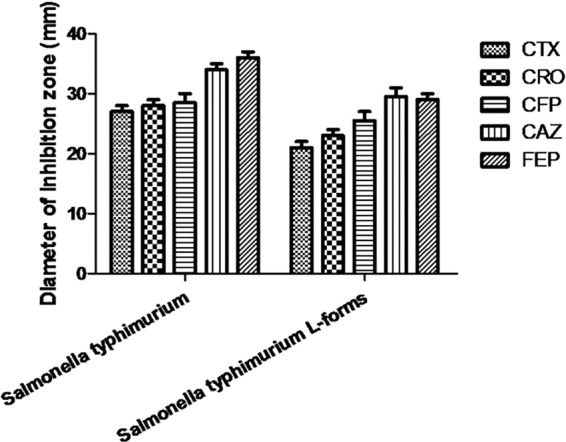
Drug susceptibility test results of *Salmonella* typhimurium and its L forms in connection with different cephalosporins Although the *Salmonella* typhimurium L forms inhibition zones were smaller than the original bacteria, there is no significant difference from the comparison of the *Salmonella* typhimurium original bacteria and L forms.

*Salmonella* typhimurium L forms colonies appeared around drug sensitive paper. The *Salmonella* typhimurium L forms colonies were tiny and gray, (shown in Fig. 2B). According to the criteria of bacterial drug susceptibility, *Salmonella typhimurium* standard strain L forms susceptibility to the third-generation cephalosporins, such as cefotaxime (CTX), ceftriaxone (CRO), cefoperazone (CFP) and ceftazidime (CAZ) remained sensitive. Salmonella typhimurium standard strain L forms’ susceptibility to the fourth generation cephalosporin cefepime (FEP) also was maintained at the level of susceptibility. Although the *Salmonella* typhimurium L forms’ inhibition zones were smaller than the original bacteria, there is no significant difference from the comparison of the *Salmonella* typhimurium original bacteria and L forms. Results are shown in Fig. 3.

#### Observation of salmonella typhimurium L form colonies

**Figure 4:**
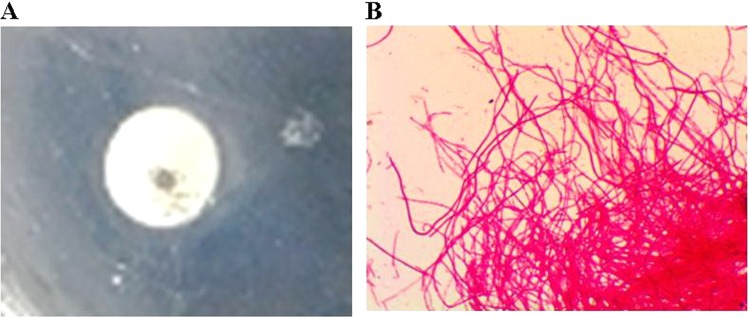
*Salmonella* typhimurium L form colonies appeared around drug sensitive paper. (**A**) *Salmonella* typhimurium L forms’ drug susceptibility test culture. The *Salmonella* typhimurium L forms colonies were tiny and gray around drug sensitive paper. (**B**) *Salmonella* typhimurium L forms Gram stain showed negative Gram stain and filamentous bacillus.

*Salmonella* typhimurium L form colonies can appear around drug sensitive paper, showed in Fig. 4A. In the L form colonies*, Salmonella* typhimurium L forms Gram stain showed negative Gram stain and filamentous bacillus, showed in Fig. 4B.

## Discussion

*Salmonella* typhimurium, commonly known as food poisoning pathogens, has many virulence factors and antibiotic resistance. It has become one of the major pathogens of nosocomial and foodborne infection and food poisoning. And it is more common in food poisoning and sepsis of infants and young children^11,12^. *Salmonella* typhimurium L forms often occur in patients after β-lactam antibiotic treatment, resulting in chronic infection. And bacterial L forms were often detected as a pathogen^13,14^. Therefore, the study on drug susceptibility of *Salmonella* typhimurium L forms is important to prevent and control *Salmonella* typhimurium and its L form infection disease.

Bacterial L-form is a cell wall-deficient type of bacteria, and bacterial cell wall peptidoglycan formation is inhibited under various factors *in vivo* and *in vitro*. But Bacterial L forms can still grow and reproduce, and they still had pathogenicity. Cephalosporins act on the bacterial cell wall to inhibit the transpeptidase activity, affect the synthesis of the peptidoglycan layer, and exert a bactericidal effect^15–17^. Cephalosporin antibiotic individuation can induce bacterial variability to L forms and result in prolonged infection in blood and bone marrow. Ceftriaxone is the third generation of cephalosporin antibiotics for the treatment of lower respiratory tract infections, skin and soft tissue infections, sepsis caused by sensitive pathogens. And similar drugs also include ceftazidime, cefotaxime and cefoperazone, as well as cefepime of the fourth generation cephalosporins^18,19^. This study found that *Salmonella typhimurium* under cephalosporin treatment can mutate to bacterial L forms, so that the drug inhibition zones of third and fourth generation cephalosporins were reduced. But *Salmonella* typhimurium L forms remained sensitive to third-generation cephalosporins including cefotaxime, ceftriaxone, cefoperazone and ceftazidime and to the fourth-generation cephalosporin cefepime, in relation to the executive standard of the national antibacterial drug susceptibility test. However, clinical medication may require extended treatment of cephalosporins by means of combination medication with other action route antibiotic, in order to achieve complete pathogen removal, including original bacteria and their bacterial L forms^20,21^.

*Salmonella* typhimurium has multi-drug resistance and can naturally resist a variety of antibacterial drugs. The main mechanisms of drug resistance are decreased permeability of the outer membrane, changes in lactamase and penicillin-binding proteins, activation and production of efflux pumps. In recent years, with the increasing number of clinically applied antibacterial drugs, broad-spectrum resistant *Salmonella* typhimurium bacteria have become more and more widespread^22–24^. Besides the influence of plasmids, the development of drug resistance depends on the type of chromosomal gene and the gene of the strain. Mutations and environmental choices are closely related. The data show that *Salmonella typhimurium* L forms still have pathogenicity. *Salmonella* typhimurium L forms have cell wall defects, so that they are more resistant to cephalosporins acting on cell walls than *Salmonella* typhimurium original bacteria. Cephalosporins alone can induce bacterial L form production, but combined use of antibiotics that act on ribosomes or nucleic acids can prevent the prolongation of disease caused by the production of L-forms. Therefore, a reasonable combination of antimicrobial agents is an effective method to kill all *Salmonella* typhimurium and reduce the *Salmonella* typhimurium L forms’ formation.

According to the antibiotic target site of pathogenic bacteria, the action mechanisms of antibiotics are divided into four categories. They inhibit bacterial cell wall synthesis, or affect cytoplasmic membrane permeability, or inhibit protein synthesis (eg. macrolides, aminoglycosides), or inhibit nucleic acid metabolism (eg. quinolone, sulfonamides). Doctors should be careful in choosing antibiotics, which should follow the principle of combination and interaction of antibiotics, in order to control *Salmonella* typhimurium infection.

## Conclusions

The antibiotic susceptibility of *Salmonella* typhimurium standard strain L forms to the third and forth generation cephalosporins remains sensitive, but its L forms inhibition zones are smaller than the original bacteria. The combined use of multi-antibiotics is a convenient and effective method to reduce *Salmonella* typhimurium L forms occurrence in order to avoid the occurrence of bacterial L form infection.

## Data Availability

The datasets used and/or analyzed during the current study available from the corresponding author on reasonable request.
